# Photochemical Method for Laser Absorption

**DOI:** 10.3390/nano12244384

**Published:** 2022-12-09

**Authors:** Weiwei Tang, Yinuo Zhang, Xingyu Qi, Yu Duanmu, Yue Yao

**Affiliations:** 1 Guangzhou Maritime University, Guangzhou 510330, China; 2 Tianjin University of Science and Technology, Tianjin 300457, China; 3 Hebei University of Technology, Tianjin 300131, China

**Keywords:** aircrafts, laser, N_2_ photofxation, Ag NPs

## Abstract

During the laser application process, laser energy is usually converted into heat energy, causing high temperature, which affects the (high-speed) aircraft in routine flight. A completely novel photochemical method was investigated to potentially minimize the energy effect of the laser beam. Ag nanoparticles/$C_{3}N_{4}$ were synthesized by an ultra-low temperature reduced deposit method with Ag mean diameters of 5–25 nm for photofixation of $N_{2}$. The absorption performance of laser can be improved by using appropriate charge density and small size Ag metal particles. The energy absorption rate was 7.1% over Ag/$C_{3}N_{4}$ (−40) at 5 $\mathrm{mJ}/{\mathrm{cm}}^{2}$ of laser energy.

## 1. Introduction

**Figure 1:**
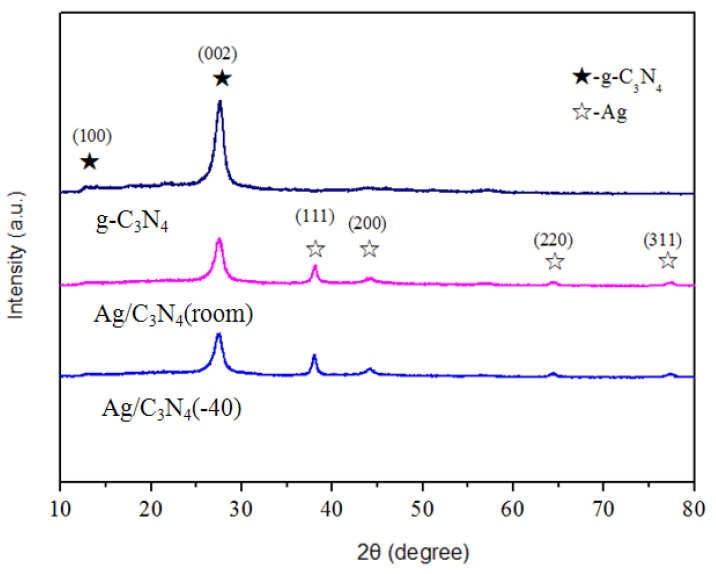
XRD of pure CN, Ag/C_3_N_4_ (−40) and Ag/C_3_N_4_ (room).

The photocatalytic $N_{2}$ reduction reaction (NRR) generating NH_3_ owing to milder conditions has attracted much attention in the field of fixation of N_2_ [1,2]. It is not only performed with high reactivity but also could be achieved under room conditions [3,4]. More importantly, the NRR photocatalyst has been improved in terms of low temperature resistance. In other words, the key to efficient N_2_ photofixation (energy transfer) is the selection of stable and high-performance catalysts, especially those prepared at low temperatures [5]. A graphitic carbon nitride (CN) supported Ag nanoparticle (NPs) catalyst is a stable photocatalyst in the range of −60 °C to 25 °C [6]. The mechanism of N_2_ photofixation reaction is shown in Figure 1. Under the light source, electrons were transferred from CN to Ag. The aggravated electron on the surface of metal NPs could promote the N_2_, and then the promoted N_2_ could be reacted with $H_{2}$ to gain ${\mathrm{NH}}_{3}$.

Proven to be the frontier of aerospace applications, high speed aircraft (HSA) for space and civilian use have increased significantly over the decades [7,8]. This results in significant interest in making safe and sustainable high-speed aircraft. However, HSA usually flies with a subsonic or supersonic speed while facing surveillance systems in most flight trajectories, such as infrared irradiation, radar wave, and laser beam risk [9]. Among them, the laser beam risk is attributed to its high energy density and high mobility, causing damage to precision navigation equipment to some extent [10]. In addition, aircraft illumination by handheld lasers, whose output ranges from 1–5 milli Watts (m W) to 5 Watts, was also a potential danger to the eyes of the pilot in the cockpit [11].

The photocatalytic reaction is usually considered to convert optical energy into stored chemical energy [12,13]. Gondal et al. [14] recently reported a chemical method for laser energy absorption using the removal of sulfur compounds from diesel. Dimethyl-dibenzothiophene (DMDBT) in diesel was deeply removed under an oxygen environment where oxidative reactions can take place by laser irradiation. Similarly and potentially, $N_{2}$ photofixation may be another way to determine the energy of laser absorption by exploring the driving force of space charge separation between Ag and CN provided by cryogenic synthesis.

Additionally, in our recent work, we found that the relation between the interfacial contact area of Ag (NPs)/CN and photocatalytic performance shows a typical volcano curve [15]. Hence, the connection of metal and the support has a significant effect on the transfer of light energy.

This paper reports a photocatalytic chemical reaction method for the synthesis of uniformly dispersed Ag decorated on CN by reducing laser energy. Compared with the Ag/CN (25 °C), the Ag/CN prepared at low temperature shows better activity, stability, and absorptive capacity under the laser condition for $N_{2}$ photofixation. As an implemented application of this paper, Ag/CN film could be a bridge between photochemical method and laser absorption to air our views. Thus, the high energy laser could be partly absorbed by releasing chemical product over Ag/CN (film) to protect the pilot and the precision navigation equipment.

## 2. Experimental 

### 2.1. Catalyst Preparation

**Scheme 1:**
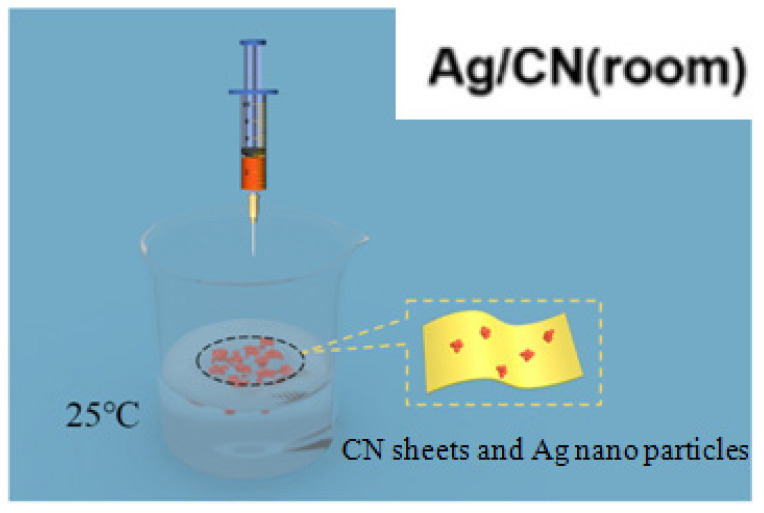
Schematic diagram of Ag/C_3_N_4_ catalysts at room temperature.

The preparation of catalysts of Ag/CN was followed as presented in the earlier report [15]. The typical synthesis was as follows: (1) For CN nanosheets, 0.5 g CN powder was added to 70 mL deionized water, stirred for 30 min, transferred to a Teflon-lined steel autoclave (Newton II–III, Shanghai LABE Instrument Co., Ltd. Shanghai, China), and heated at 180 °C (12 h). Then, the CN nanosheets were obtained after centrifugation and drying. (2) For Ag/CN (room), $C_{3}N_{4}\;$(1.0 g) was dispersed in methanol (50 mL) (named Solution A). The dispersed ${\mathrm{AgNO}}_{3}$ solution (1 mol/L) was prepared by an ultra-low-temperature process at different temperatures (named Solution B). After cooling, solution B was added to solution A for 30 min and reacted entirely for another 3 h. The obtained mixture was centrifuged and dried at 30 °C for 8 h. The Ag/CN photocatalysts were labeled as Ag/CN(X), X stands for the reduced temperature (−40 °C and 25 °C (RT)) (see the synthesis in Scheme 1 and Picture S1).

### 2.2. Characterization

The materials were characterized by XRD, SEM, TEM, etc. The descriptions of the characterization are in the Characterization part of the Supplementary Material.

### 2.3. Electrochemical Measurements and Computational Details

Electrochemical measurements were performed on a CHI 660D electrochemical workstation. Spin polarization calculations were performed using VASP (Vienna ab initio simulation package) code on DFT (density functional theory). See Supplementary Materials.

### 2.4. Photocatalytic Performance with Laser

**Scheme 2:**
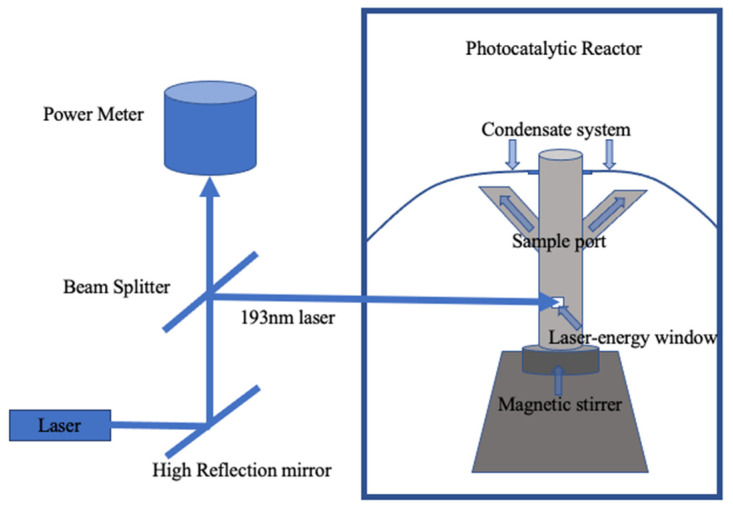
The evaluation device.

A method and a setup based on a chemical reaction have been developed to absorb synthetic ammonia under laser irradiation. The reactor (Scheme 2) consists of a double-walled quartz photocatalytic vessel of 500 mL volume with another quartz window for transmission of the laser beams. A total of 0.1 g of photo-catalyst mixed with 200 mL solution (ammonia absorption) of 5% alcohols was irradiated by a laser lamp (ArF laser). The laser energy range studied was between 50 and 200$\;\mathrm{mJ}/{\mathrm{cm}}^{2}$ [16]. A specific volume of the upper solution was periodically sampled by pipette. The concentration of product ${\mathrm{NH}}_{3}$ was measured by Nessler’s reagent spectrophotometry method (Cary-50, Varian Co., Shanghai, China) [16].

## 3. Results and Discussion

### 3.1. XRD

All samples exhibited two diffraction peaks located at nearly 13° and 27.5°, as seen in Figure 1. These peaks at 13° and 27.5° are denoted as the melon structure and crystal plane (100) of CN, respectively [17]. The intensity of the CN diffraction peaks at 13° and 27.5° obviously decreased with the addition of Ag at different temperatures for preparation. Diffraction peaks of Ag were located at 38°, 44°, 64.5°, and 77.5°. The oxide state could not be determined.

### 3.2. The Morphology of Ag/CN (−40)

**Figure 2:**
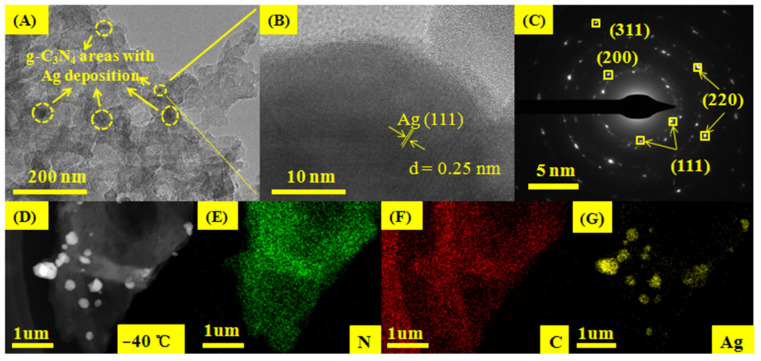
TEM images and its element distribution for Ag/C_3_N_4_ (−40). (**A**–**C**) TEM images, (**D**) HAADF-STEM image, (**E**–**G**) element distribution of N, C, and Ag.

Figure 2 displays the TEM images and elements distribution of N, C, and Ag of Ag/$C_{3}N_{4}\left(-40\right)$. Facet Ag (111) can be observed clearly on the surface of g-$C_{3}N_{4}$ and can be proved by HAADF and SAED images. The uniform distribution of Ag nanoparticles (NPs) with a uniform size range can be observed in Figure S1 (with an average size of 13 nm). The detailed microstructure of Ag/CN (−40) was further investigated by the three elements distribution mapping [8,18,19]. The results are shown in Figure 2E–G; the Ag NPs were finely loaded not only onto the outer surface but also onto the inner surface of CN. According to the element distribution mapping, the ratio of Ag surface area and the surface area of support was near 1:10 (yellow dots:(green dots + red dots)), that can be used to estimate the area ratio of Ag NPs to the BET surface area of Ag/C_3_N_4_ (−40).

### 3.3. Energy Absorption Calculation

#### 3.3.1. Analysis of the Energy Gaps of Ag/CN

**Figure 3:**
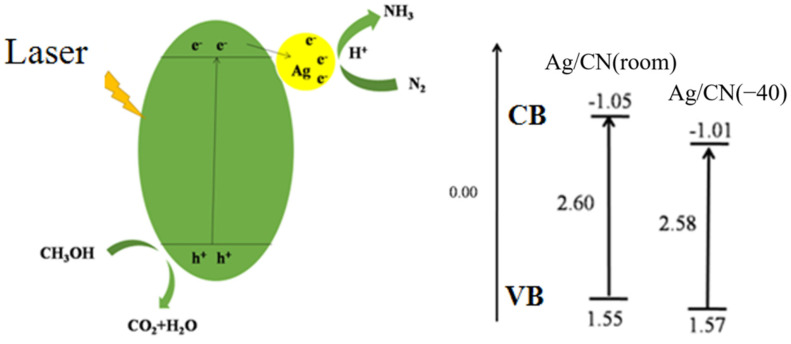
Schematic diagram of the band structure over Ag/CN for N_2_ photofixation.

When incident light irradiated on the CN (Ag/CN) and the band gap energy could be exceeded, photo-generate $e^{-}$ in VB could jump to reach CB. At the same time, $h^{+}$ is left on VB with strong activity. The Ag species are usually considered as an electron sink. $e^{-}$ generated in CB of CN would be transferred to VB by the Ag species. Energy is transferred with the charge transfer. Then $N_{2}$ could be reduced by the accepted $e^{-}$ to be the ${\mathrm{NH}}_{3}$ product. The reaction mechanism for $N_{2}$ photofixation over Ag/CN is shown in Figure 3. The flat band potentials of Ag/CN (room) and Ag/CN (−40) are −1.05 and −1.01 eV. The energy gap between VB and CB of the two catalysts is −2.60 and −2.58 eV (see the calculated band gap energy in Supplementary Material) [8].

#### 3.3.2. Analysis of the Charge Density

**Figure 4:**
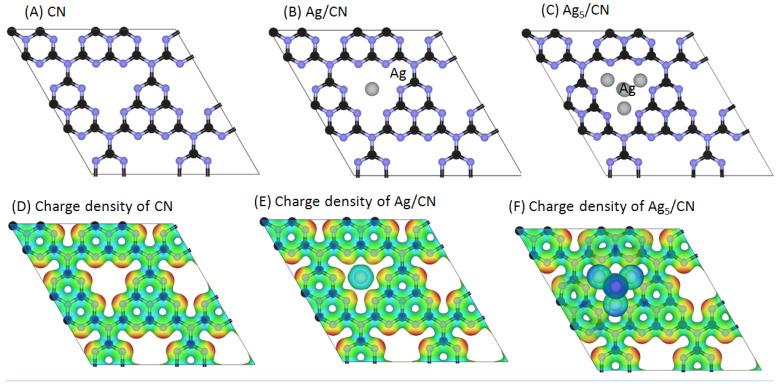
The adsorption configurations of (**A**) CN; (**B**) Ag/CN; (**C**) Ag_5_/CN and the simulation of charge density of (**D**) CN; (**E**) Ag/CN and (**F**) Ag_5_/CN.

In Figure 4A–C, the adsorption configurations of CN, ${\mathrm{Ag}}_{1}$/CN, and ${\mathrm{Ag}}_{5}$/CN are simulated by DFT [20,21,22,23]. ${\mathrm{Ag}}_{1}$ and ${\mathrm{Ag}}_{5}$ both exist as stable with the adsorption energies of −1.57 and −0.60 eV for each Ag atom, respectively. The results of Figure 4D–F show that the electron transfer pathway should be described by charge around the Ag atom (all of the Ag atoms showed cyan, which means the account was depleted by transfer). The dark cyan in Figure 4F of the Ag atom displayed abundant electron transfer. ${\mathrm{Ag}}_{5}$ supplies more electron transfer than CN and ${\mathrm{Ag}}_{1}$. The density of states (DOS) of Ag_5_–CN to simulate the electron transfer to $N_{2}$ measured in Figure S2. Ag_5_ NPs were considered as the most efficient energy transfer dot in this photocatalyst.

#### 3.3.3. Activity

**Figure 5:**
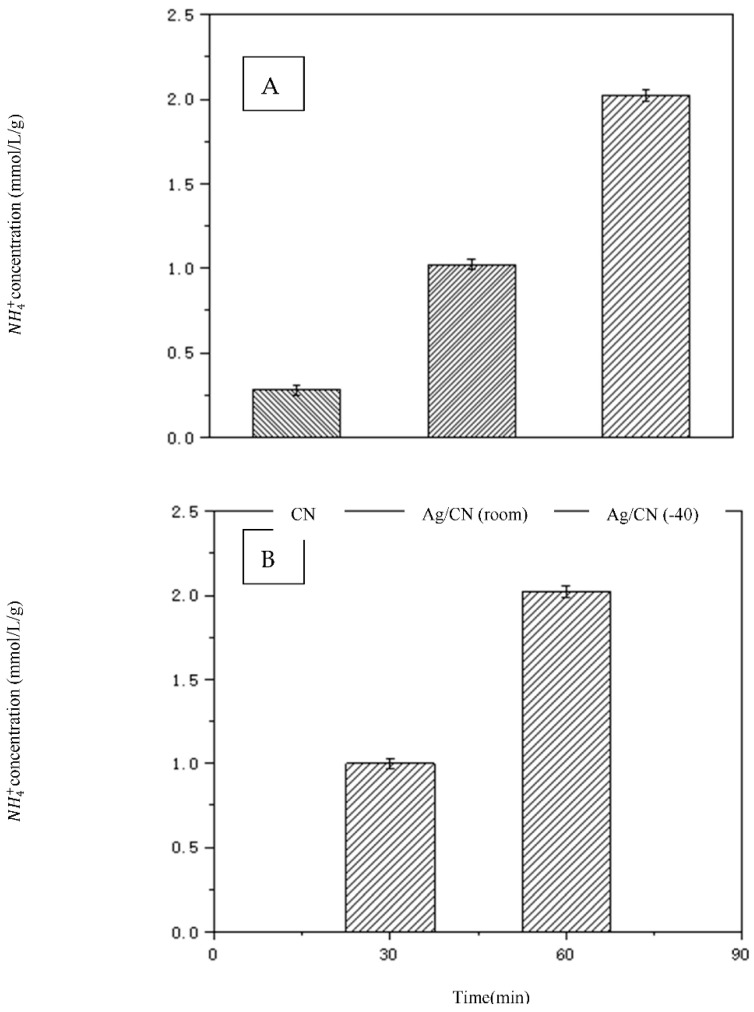
(**A**) N_2_ fixation rate in 1 h. (**B**) N_2_ fixation rate of Ag/CN (−40) at different times. The error bars are according to the test error of Nessler’s reagent spectrophotometer method.

The activity of the $N_{2}$ photofixation was displayed in Figure 5. The Ag/CN (room) catalyst can achieve $N_{2}$-fixation rate (NH_4_^+^ concentration) of 1.02 mmol/L/h/g. In contrast, this value is almost half that of Ag/CN (−40). The N_2_ fixation rate of Ag/CN (−40) was 0, 1.00, and 2.02 mmol/L/h/g at 0 min, 30 min, and 60 min, respectively. The better N_2_ photofixation effect were performed over Ag/C_3_N_4_ (−40) photocatalyst.

### 3.4. Relation between Lifetime, Charge Density, and Energy Absorption

Table 1 shows the electronic properties of Ag/$C_{3}N_{4}$ for Ag particle size, lifetime, and charge density. The particle size of Ag increases significantly (from 13.2 nm to 33.2 nm) with the temperature changing to 25 °C. The photoelectron lifetime (τ) of Ag/CN is calculated by 1/(2π$f_{\mathrm{peak}}$) [24,25,26]. $f_{\mathrm{peak}}$ (the maximum frequency peak) explains that the charge density ${\mathrm{Ag}}_{5}$ is more than ${\mathrm{Ag}}_{1}$ for the opportunity of increased charge recombination. There is reasonable concordance between the Ag adsorption configuration over CN (in Figure 4) and lifetime. The laser energy absorption rate is also calculated using the formula below (Table 1). $N_{2}$ photofixation energy was calculated by the formula in Scheme S1. The corresponding energy absorption rate was 7.1% over Ag/$C_{3}N_{4}$(−40) [19]. It should also be noted that the minimum energy value to excite the photoelectrons is equal to the value of the energy gap between VB and CB of the catalysts. The photo fixation energy transferred from the laser, which could be simulated with each Ag NP, is probably higher.

Based on the above discussion, considering the better performance over Ag/CN (−40) for N_2_ photofixation, Ag_5_ NPs were proven as the most efficient energy transfer dots in the charge density model. The laser energy absorption through the photocatalytic way can be explained by two points: (1) The enhanced photocatalytic activities over Ag/$C_{3}N_{4}$ can be driven by the laser energy. The driving energy of $\;N_{2\;}$ photofixation is transferred by the Ag NPs as a photo-generated charge. (2) The particle size affected the Ag NPs of Ag/$C_{3}N_{4}$ to contact closely, and ${\mathrm{Ag}}_{5}$ can improve the charge-separation and transfer efficiently.

## 4. Conclusions

In summary, as an efficient $N_{2}$ photofixation light source, the laser beam energy was absorbed and consumed through this chemical reaction. The excited photo-generated charge by laser energy was transferred by ${\mathrm{Ag}}_{5}$ with high charge density to produce ${\mathrm{NH}}_{3}$. The Ag/CN (−40) exhibits remarkable activity with an ${\mathrm{NH}}_{3}$ conversion of 2.02 mmol/L/h/g (gas products at room conditions, which could be the result of simple separation with the HSA), which is about double the improvement compared to that of Ag/CN (room). It also shows a favorable energy absorption rate during this photochemical method for the laser absorption process.

## Figures and Tables

**Table 1:** The properties of Ag/C_3_N_4_.

Sample	Ag Particle Size (nm)	Life Time [15] (τ, ms)	Charge Density	BET Surface Area (m^2^/g)	Energy ^a^ Absorption Rate (%)
Ag/C_3_N_4_ (−40)	~13.0	0.29	${\mathrm{Ag}}_{5\;}$	12	7.1
Ag/C_3_N_4_ (room)	~33.2	0.20	${\mathrm{Ag}}_{1}$	13	1.2

^a^: energy absorbed by N_2_ photofixation/ energy of the laser.

## Data Availability

Not applicable.

## Supplementary Materials

The following supporting information can be downloaded at: https://www.mdpi.com/article/10.3390/nano12244384/s1, Picture S1 Constant Temperature Water-bathing (EYELA PSL-1810). Figure S1: TEM image (A) and its corresponding particle size distribution (B) for Ag/C_3_N_4_ (−40); Figure S2. DOS of (A) free N_2_ (B) N_2_-Ag/CN and (C) N_2_-Ag_5_/CN.; Figure S3. EIS changes for the samples; Table S1: The effect of Ag nanoparticle size for electronic properties; Scheme S1: Calculation method.
