# Supplementary material for: Photochemical Method for Laser Absorption
Source: Nanomaterials (Basel). 2022 Dec 9;12(24):4384. doi: 10.3390/nano12244384 (PMC9783274; doi:10.3390/nano12244384)
Supplement: Supplementary file 1 [file nanomaterials-12-04384-s001.zip › nanomaterials-2071438-supplementary.pdf]

Supplementary Materials

Photochemical Method for Laser Absorption

2.2. Characterization

The crystal phase was determined by Bruker D8 CEVANCE using Cu-Kα radiation. Transmission electron microscopy (TEM, including mapping) images were obtained at room temperature on a Tecnai G2 F20 field-emission transmitting electron microscope (Philips) operating at 30kV. The BET surface area was determined by a physical adsorption instrument (Quantachrome Instruments, Autosorb-IQ).

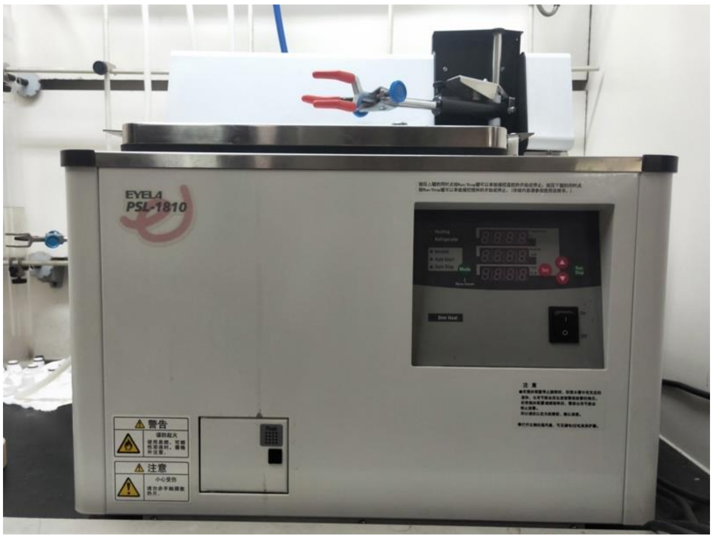

Picture. S1 Constant Temperature Water-bathing (EYELA PSL-1810).

Constant Temperature Water-bathing can easily be controlled the temperature at 25 ℃, and -40 ℃ with stirring for 1- 4 h. It adopts double compressors and strong refrigeration to achieve the required temperature faster. The temperature range is -80 ℃ -0 ℃. And we can easily control the temperature at -40 ℃ by stirring for 4 h.

2.3. Electrochemical measurements and Computational details

Electrochemical measurements were performed on a CHI 660D electrochemical workstation (Shanghai Chenhua, China) using a standard three-electrode cell with a working electrode, a standard Ag/AgCl electrode as a reference electrode, and a Pt electrode as the counter electrode. The preparation of working electrode: samples were dispersed in water to form a 5 mg ·mL^{-1} solution and ultrasonicated for 20 min; 0.1 mL of solution was dropped on the FTO glass and dry at room temperature. Spin-polarization calculations were performed using VASP (Vienna ab initio simulation package) code on DFT (density functional theory). The PBE (Perdew-Burke Ernzerhoff) was adopted to treat the electron transfer effects by GGA (generalized gradient approximation). The DFT-D3 methodology, cutoff energy of 450 eV, and a 3 × 3 × 1 Monkhorst-Pack grid were simulated by PBE. The optimized structures were obtained with the criteria of < 0.03 eV/Å in force and 10^{-5} eV in energy. Valence electrons: C $2s^22p^2$, N $2s^22p^3$, and Ag $4d^{10}5s^1$.

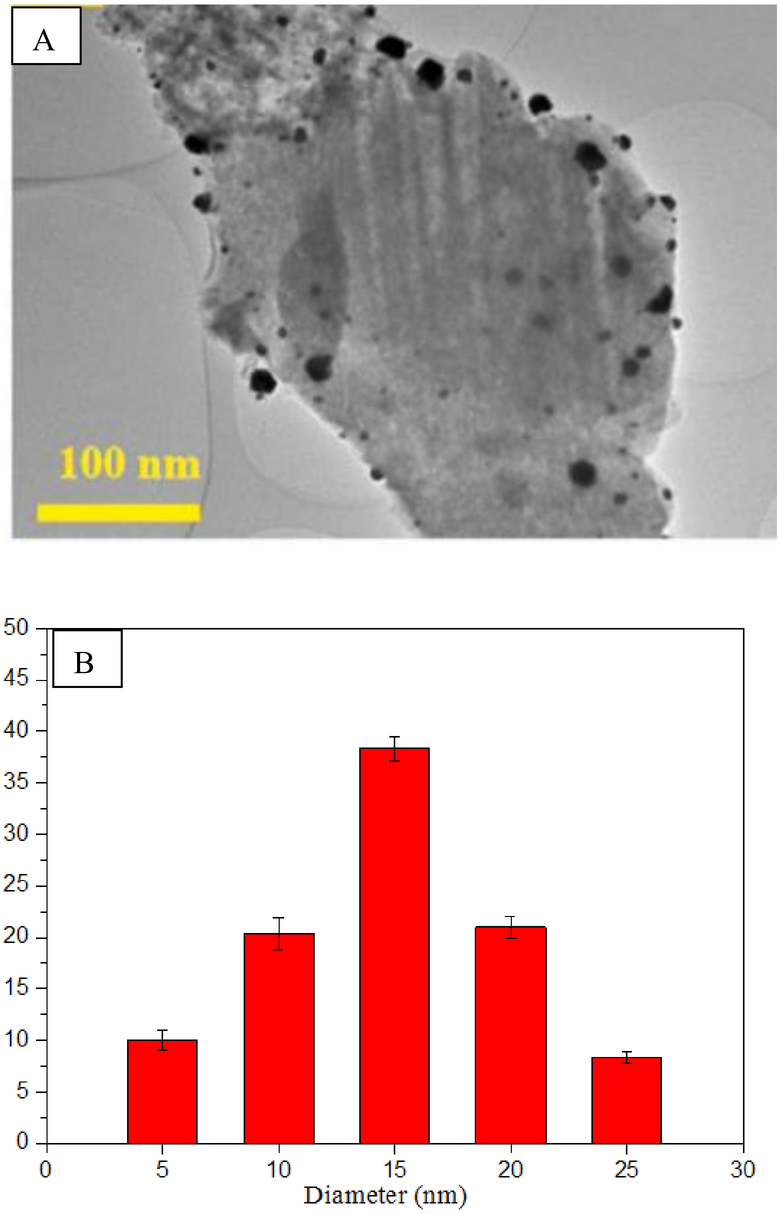

Figure. S1. TEM image (A) and its corresponding particle size distribution (B) for Ag/C_3N_4(- 40)

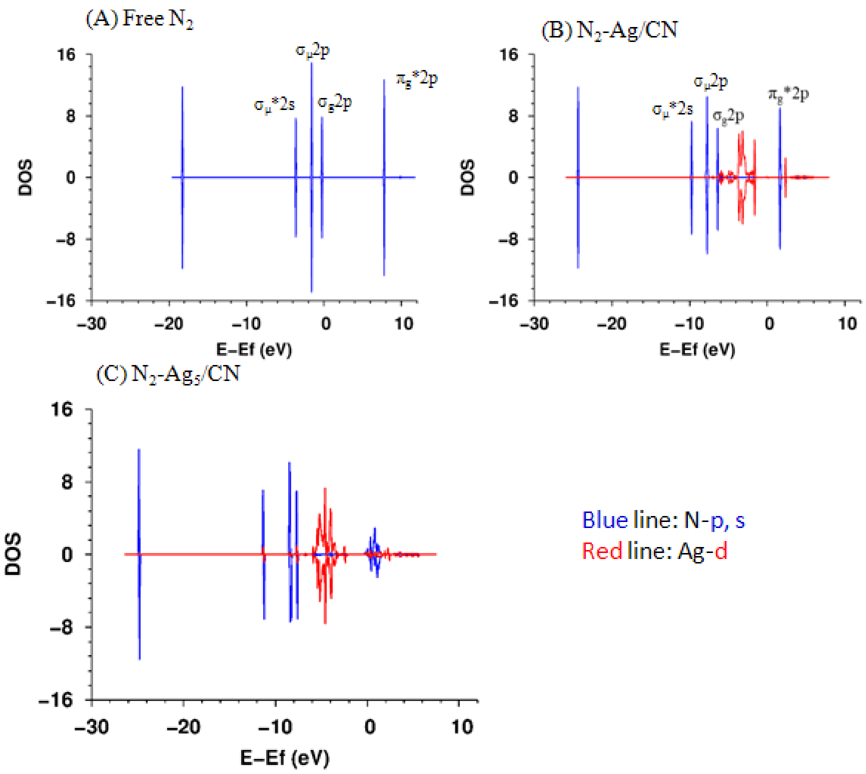

Figure. S2. DOS of (A) free N2 (B) N2-Ag/CN and (C) N2-Ag5/CN.

The density of states (DOS) of free N_2, adsorbed on two kinds of Ag_5-CN, improves the electron transfer between the Ag and N_2. There are four peaks near the Fermi level in the control blank sample in Fig.S2(A). In Fig.S2(B) and (C), the π_g*2p orbit moves towards the Fermi level, and the σ_g2p orbit moves away from the Fermi level, indicating an increase in the number of electrons in the π_g*2p orbit over all of the two samples. Additionally, the σ_g2p orbit is hybridized with the d orbit of Ag, and there is an obvious overlap of state density peaks near -6.3 eV and 1.1 eV, indicating that Ag atoms transfer electrons to the π_u2p orbit of N through the σ_g2p orbit. The π_g*2p orbit of N and d orbit of Ag also undergo strong hybridization, indicating the electron transfer between Ag and N_2.

Calculated band gap energy

The valence band (E_{VB}) and conduction band (E_{CB}) position of the samples were calculated on the follow equation:

$E_{VB} = X - E^e + 0.5\ E_g$

$E_{CB} = E_{VB} - E_g$

Where X is the electronegativity of CN (4.73 eV vs. NHE), E^e is the free electron energy on the hydrgoen scale (~ 4.5 eV vs. NHE). The corresponding estimated band gap energy E_g are calculated and given in Fig 3.

Table. S1 The effect of Ag nanoparticle size for electronic properties.

Sample	Ag particle size (nm) [a]	Work function (φ, eV) [b]	SBH (eV) [c]	lifetime (τ, ms)
C_3N_4	-	4.62		0.17
Ag/C_3N_4(-40)	~13.2	4.82	0.20	0.29
Ag/C_3N_4(-60)	~9.2	4.86	0.24	0.24

[a]From TEM.
[b]$\varphi = \varphi_b + 1.08/d_P$, φ_b is the work function of bulk Ag (4.74 eV), d_P is the Ag particle size; the work function of C_3N_4 is 4.62 eV (obtained from DFT calculation).
[c]Schottky barrier height (SBH), SBH =$\varphi - x$, where x is the work function of C_3N_4 (4.62 eV).

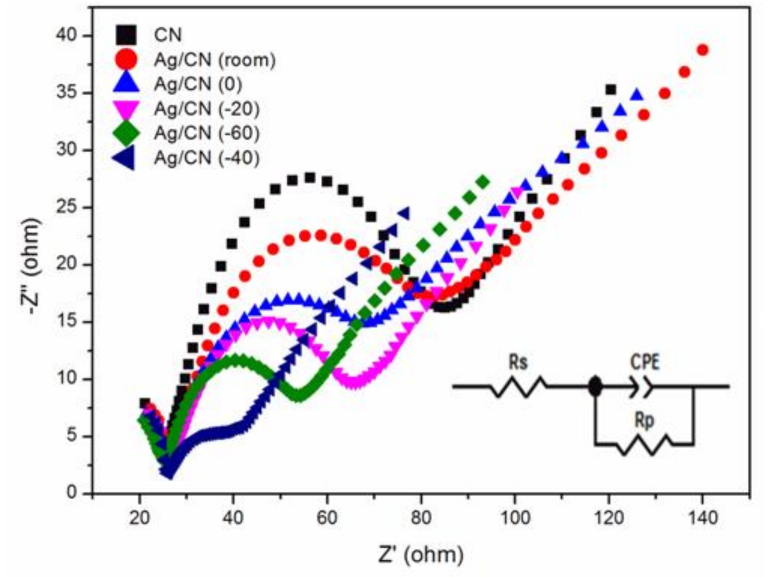

Figure. S3. EIS changes for the samples.

$$\eta = \left(B/A\right) \times 100\%$$
$$A = A_0 \cdot S_A$$
$$B = \frac{n \cdot h}{S_0}$$
$$h = 5 \cdot h_0$$
$$n = \frac{S}{S_{Ag_5}}$$
$$S = \alpha \cdot S_{BET} \cdot \omega$$
$$S_{Ag_5} = \pi\left(d/2\right)^2$$

Scheme S1 calculation method

η : Energy absorption rate, %

A : Energy of laser, mJ

A_0 : Laser incident energy per unit area,5mJ/cm^2 [14]

S_A : The area of the laser-energy window，0.01cm×0.01cm

B : Energy absorbed by N_2 photofixation, mJ

h : Energy transferred by Ag_5 of Ag/C_3N_4(-40), mJ

h_0: Energy transferred by per Ag atom of Ag/C_3N_4(-40), mJ（1eV=1.6 $\times 10^{-16} mJ$ ）

n : The number of Ag_5

S : The total surface area of Ag_5, mJ/cm^2

α : The area ratio of Ag_5 to the BET surface area of Ag/C_3N_4(-40), 0.1(calculated by TEM mapping)

S_{BET}: The BET surface area of Ag/C_3N_4(-40), 1.2· $10^5 cm^2/g(12m^2/g)$

ω : The average unit mass of catalyst for TEM mapping, 0.001g

S_{Ag_5}: The surface area of each Ag_5, cm^2

d : The diameter of Ag NPs, cm
